# Injectable rhBMP-2-loaded calcium phosphate cement and chitosan composite hydrogel for the repair of osteoporotic bone defects

**DOI:** 10.1186/s12891-026-09717-w

**Published:** 2026-03-09

**Authors:** Minglei Cai, Jian Huang, Fulong Zhong, Bowen Lai, Jing Wang, Tielong Liu

**Affiliations:** 1 Department of Orthopaedic Oncology, The Second Affiliated Hospital of Naval Medical University, No. 415 Fengyang Road, Huangpu District, Shanghai, 200003 China; 2 Department of Orthopaedics, The Second Affiliated Hospital of Naval Medical University, No. 415 Fengyang Road, Huangpu District, Shanghai, 200003 China; 3 School of Clinical Medicine, Shandong Second Medical University, No. 7166 Baotong West Street, Weifang, 261053 China; 4 Department of Orthopaedic Surgery, Zhongshan Hospital, Fudan University, No.180 Fenglin Road, Shanghai, 200032 China

**Keywords:** Osteoporosis, rhBMP-2, Chitosan, Calcium phosphate cement, Hydrogel, Bone regeneration, Bone tissue engineering

## Abstract

**Background:**

Regeneration of osteoporotic bone defects is a major clinical challenge due to impaired osteogenic activity and high infection risks. We developed an injectable rhBMP-2-loaded calcium phosphate cement (CPC) and chitosan (CH) composite hydrogel (rhBMP-2/CPC@CH) to evaluate its therapeutic efficacy for repairing osteoporotic bone defects.

**Methods:**

The rhBMP-2/CPC@CH hydrogel was synthesized and characterized for its physicochemical properties and enzymatic degradation. Its osteogenic potential was assessed using BMSCs. An OVX-induced rat cranial defect model was established to evaluate in vivo bone repair via Micro-CT and histological analysis. Antibacterial activity and effects on wound healing were also investigated.

**Results:**

The rhBMP-2/CPC@CH hydrogel exhibited excellent injectability, self-healing, and enzymatic degradation properties with a uniform elemental distribution. The hydrogel significantly promoted the osteogenic differentiation of BMSCs, as evidenced by increased ALP activity and mineralized nodule formation. In the OVX-induced rat model, Micro-CT and histological analysis demonstrated that the hydrogel significantly increased bone mineral density and bone volume fraction, leading to dense new bone formation. Furthermore, the hydrogel effectively inhibited the growth of *S. aureus* and *E. coli* without adverse effects on wound healing, establishing a protected regenerative microenvironment.

**Conclusions:**

The rhBMP-2/CPC@CH hydrogel accelerates the repair of osteoporotic bone defects by providing an osteoinductive microenvironment and intrinsic antibacterial defense, representing a promising minimally invasive strategy for clinical bone regeneration.

**Supplementary Information:**

The online version contains supplementary material available at 10.1186/s12891-026-09717-w.

## Background

Osteoporosis (OP), a systemic metabolic bone disease, is characterized by reduced bone mass and microarchitectural deterioration, leading to a significantly increased risk of fragility fractures [1]. Among the elderly population, osteoporotic fractures are prevalent, particularly in the vertebrae and hip, often resulting in severe disability and high mortality rates [2, 3]. Currently, the primary methods for treating osteoporotic bone defects include open surgery, long-term pharmacological therapy, and minimally invasive interventions [4]. However, open surgery is associated with risks such as reduced fixation stability and secondary fractures [5]. Long-term drug therapy carries risks of adverse effects such as osteonecrosis of the jaw (ONJ) and atypical femoral fractures (AFF) [6–8], while autografts in osteoporotic patients are limited by poor bone quality and donor site morbidity. Therefore, the quest for novel, minimally invasive, and bioactive materials capable of effectively reconstructing osteoporotic bone defects is imperative to enhance clinical outcomes for affected individuals.

Biomaterials have been employed in fabricating hydrogels for bone tissue engineering, showcasing notable progress. Calcium phosphate cement (CPC), a widely used bone substitute, exhibits exceptional osteoconductivity and injectability, providing a scaffold for mineral deposition [9]. Recombinant human bone morphogenetic protein-2 (rhBMP-2), a potent osteoinductive factor, possesses the ability to drive mesenchymal stem cell differentiation and accelerate bone formation via the Smad signaling pathway [10, 11]. Chitosan (CH), a natural cationic polysaccharide, offers biocompatibility, biodegradability, and intrinsic antibacterial properties, effectively inhibiting bacterial infections that may occur during the bone repair process [12, 13]. Consequently, the fabrication of an injectable composite system presents a promising avenue for minimally invasive adaptation to irregular defects. The rhBMP-2/CPC@CH hydrogel is designed to leverage the complementary advantages of these components, achieving a balance between mechanical stability and sustained bioactive release. This rational design highlights its potential to facilitate the regeneration of osteoporotic bone defects while minimizing the risk of infection [14, 15].

However, bone repair in osteoporotic patients is significantly challenged by impaired osteogenic activity and a heightened susceptibility to infection. Accordingly, there is an urgent need for a dual-functional system capable of providing both potent osteoinductive signaling and robust antibacterial protection to ensure successful regeneration in such a pathological microenvironment. This study aimed to investigate the therapeutic efficacy of the injectable rhBMP-2/CPC@CH composite hydrogel for treating osteoporotic bone defects in a rat model. The assessment encompassed physicochemical characterization, in vitro biocompatibility and osteogenic differentiation, in vivo bone regeneration in an ovariectomized (OVX) cranial defect model, and antibacterial performance against *S. aureus* and *E. coli*. The objective of this study was to verify the repair effect of this multifunctional composite system in an osteoporotic environment, thereby laying an experimental foundation and providing methodological support for future clinical trial design and application.

## Methods

### Preparation of injectable rhBMP-2/CPC@CH composite hydrogel

Recombinant human bone morphogenetic protein-2–loaded calcium phosphate cement (rhBMP-2/CPC) granules were provided by Shanghai Rebone Biomaterials Co., Ltd. (Shanghai, China). Medical-grade chitosan (CH) gel approved for clinical application was purchased from Changsha Hairun Biotechnology Co., Ltd. (Changsha, China) and used as received. RhBMP-2/CPC artificial bone blocks were mechanically crushed into granular particles and used as osteoconductive carriers. The CPC granules, comprising primarily α-tricalcium phosphate and hydroxyapatite, were gradually incorporated into pre-prepared chitosan gel at room temperature under continuous magnetic stirring to form a stable, injectable paste rather than a traditional hardened cement block, ensuring uniform dispersion and preventing aggregation. The mixing process was carefully controlled to maintain suitable viscosity and injectability while minimizing air entrapment. The composite formulation was adjusted to achieve a rhBMP-2 content of approximately 0.25 mg per 1 mL of the final hydrogel. Pure chitosan gel prepared using the same procedure served as the CH control. All preparation procedures were conducted under aseptic conditions. To maintain biological activity, the prepared composite components were stored at 4℃ until use.

### Morphological and chemical characterization

Surface morphology of CH and rhBMP-2/CPC@CH hydrogels was examined by scanning electron microscopy (SEM). Samples were dried, mounted on aluminum stubs, and sputter-coated with gold. SEM observation was performed at an accelerating voltage of 15 kV to evaluate microstructural features and CPC particle distribution within the chitosan matrix. Elemental composition and spatial distribution of calcium (Ca) and phosphorus (P) were analyzed using energy-dispersive spectroscopy (EDS) coupled with SEM to assess the dispersion of CPC-derived inorganic components. Chemical composition was characterized by Fourier transform infrared spectroscopy (FTIR). Dried samples were analyzed over a wavenumber range of 4000–400 cm⁻¹ to confirm the coexistence of chitosan and CPC components within the composite hydrogel.

### Rheological properties

Rheological properties of the rhBMP-2/CPC@CH hydrogel were measured using a rotational rheometer (Anton Paar MCR92, Austria) equipped with parallel plates at 37 °C. Hydrogel samples were placed on the lower plate, and the gap was adjusted to ensure uniform sample distribution. An amplitude sweep test (0.01–100% strain, 1 Hz) was conducted to determine the linear viscoelastic region. Frequency sweep measurements (0.1–100 Hz) were then performed within this region to record storage modulus (*G’*) and loss modulus (*G’’*). Shear rate–dependent viscosity was measured over a range of 0.1–1000 s⁻¹ to evaluate shear-thinning behavior and injectability.

### Enzymatic degradation assay

Enzymatic degradation of CH and rhBMP-2/CPC@CH hydrogels was evaluated using a chitosanase-mediated degradation model. Initial sample weights were recorded as *W*_*0*_, and samples were immersed in a 900 U/L chitosanase solution and incubated at 37 °C. At predetermined time points, samples were collected, gently blotted, and weighed to obtain the remaining weight (*W*_*1*_). The remaining weight% was calculated as:

$$Remaining\:weight\:\left(\%\right)=\left(W_{1}/W_{0}\right)^{*}100\%$$

degradation behavior was determined based on changes in remaining weight over time.

### Cell culture

Bone marrow mesenchymal stem cells (BMSCs) were isolated from 3-week-old female Sprague–Dawley rats. For indirect coculture experiments, CH and rhBMP-2/CPC@CH hydrogels were immersed in complete culture medium and incubated at 37 °C for 24 h to obtain hydrogel extracts, which were filtered and stored at 4 °C. BMSCs were cultured in low-glucose DMEM supplemented with 10% fetal bovine serum and 1% penicillin–streptomycin at 37 °C with 5% CO₂. Medium was refreshed every 3 days, and passage 3 cells were used for all experiments.

### Cytocompatibility assays

Cytocompatibility of the hydrogels was assessed by Live/Dead staining and CCK-8 assays. For Live/Dead staining, hydrogels were injected into 6-well plates, sterilized by ultraviolet irradiation, and seeded with BMSCs at 5 × 10⁴ cells per well. After 24 h, cells were stained with calcein-AM (2 µM) and propidium iodide (4.5 µM) using a Live/Dead staining kit (Yeasen Biotechnology, Shanghai, China) and observed under a fluorescence microscope (Leica, Germany). Cell proliferation was evaluated using a CCK-8 assay (Yeasen Biotechnology, Shanghai, China). BMSCs (5 × 10³ cells per well) were cultured on hydrogels in 96-well plates for 1 and 4 days, followed by absorbance measurement at 450 nm using a microplate reader (BioTek, USA).

### Alkaline phosphatase (ALP) activity assay

Hydrogel extracts were prepared by immersing CH and rhBMP-2/CPC@CH hydrogels in basal low-glucose DMEM for 48 h and supplemented with osteogenic inducers (10 mM β-glycerophosphate, 50 µM ascorbic acid, and 100 nM dexamethasone). BMSCs (1 × 10⁵ cells per well) were cultured in 6-well plates and treated with the corresponding conditioned medium. After 14 days, ALP staining was performed using a BCIP/NBT kit (Beyotime Biotechnology, Shanghai, China). ALP activity was quantified using an ALP activity assay kit (Qiyuan Biotechnology, Shanghai, China) and normalized to total protein content.

### Alizarin red S (ARS) staining

Extracellular matrix mineralization was evaluated by Alizarin Red S (ARS) staining. BMSCs (1 × 10⁵ cells per well) were cultured in hydrogel-conditioned osteogenic medium for 21 days, fixed with 4% paraformaldehyde, and stained with ARS solution (Solarbio, Beijing, China) for 30 min. After removal of excess dye, mineralized nodules were observed and imaged using an optical microscope (Leica, Germany).

### Quantitative real-time PCR (RT-qPCR) analysis

The expression of osteogenesis-related genes was analyzed by quantitative real-time polymerase chain reaction (RT-qPCR). Total RNA was extracted from BMSCs using a SteadyPure Universal RNA Extraction Kit II (Accurate Biotechnology, China), and cDNA was synthesized using an Evo M-MLV reverse transcription kit (Accurate Biotechnology, China) according to the manufacturers’ instructions. RT-qPCR was performed using SYBR Green SupTaq HS Premix (Accurate Biotechnology, China). The expression levels of *ALP*,* RUNX2*,* and OCN* were normalized to β-actin and calculated using the 2^⁻ΔΔCt^ method. Primer sequences are listed in the Table 1.

**Table 1:** Primer sequences used for RT-qPCR analysis

Gene	Primer Sequence (5’–3’)
ALP	F:5’-AGGCATAGACTTCAACCAGCC-3’ R:5’-ACTCCGTACCAAAGCCATCG-3’
RUNX-2	F:5’-AGGCAGGTGCTTCAGAACTGG-3’ R:5’-GCATTCGTGGGTTGGAGAAGC-3’
OCN	F:5’-CTGACAAAGCCTTCATGTCCAAG-3’ R:5’-TCCAAGTCCATTGTTGAGGTAGC-3’
β-actin	F:5’-CTAGGCACCAGGGTGTGATG-3’ R:5’-AGGTCTCAAACATGATCTGGGT-3’

### Western blot analysis

Protein expression of osteogenesis-related markers was analyzed by Western blot. Total protein was extracted from BMSCs using RIPA lysis buffer (Servicebio, Wuhan, China) supplemented with protease inhibitors, and protein concentration was determined using a BCA protein assay kit (Beyotime Biotechnology, Shanghai, China). Equal amounts of protein were separated by SDS–PAGE and transferred onto PVDF membranes (Servicebio, Wuhan, China). After blocking, membranes were incubated with primary antibodies against ALP, RUNX2, and OCN (Immunoway, USA), followed by incubation with corresponding secondary antibodies. Protein bands were visualized using an enhanced chemiluminescence (ECL) detection kit (Servicebio, Wuhan, China), and β-actin was used as the internal loading control.

### In vivo cranial defect model and hydrogel implantation

A rat cranial defect model was established to evaluate the osteogenic efficacy of injectable hydrogels in osteoporotic bone regeneration. All animal procedures were approved by the Animal Research Ethics Committee of Naval Medical University [NMUE 2024-026] and conducted in accordance with relevant guidelines. Six-month-old female Sprague–Dawley rats were purchased from Shanghai BK Bio-Science Co., Ltd. (Shanghai, China) and acclimated prior to surgery. Osteoporosis was induced by bilateral ovariectomy (OVX). After 6 weeks of osteoporosis development, OVX rats were used to establish a critical-sized cranial defect model. Anesthesia was induced via intraperitoneal injection of sodium pentobarbital (40–50 mg/kg). A total of 18 osteoporotic rats were randomly assigned using a random number table into three groups (*n* = 6 per group): Control group, CH group, and rhBMP-2/CPC@CH group. Following anesthesia, a circular cranial defect with a diameter of 5 mm was carefully created in the parietal bone. For the treatment groups, hydrogels were injected directly into the defect site at a volume of 40 µL per defect. The incision was subsequently closed in layers. All surgical procedures were well tolerated, and post-operative analgesia was provided to minimize distress. All animals remained in good health throughout the study, and no animals were excluded from the final analysis due to infection or unintended death. At the end of the experimental period, all rats were euthanized using an overdose of sodium pentobarbital (150 mg/kg, i.p.) under deep anesthesia.

### Micro-CT analysis

Micro–computed tomography (micro-CT) was used to assess in vivo bone regeneration within the cranial defect sites. At 4 and 8 weeks post-implantation, rats were euthanized, and calvarial specimens containing the defect region were harvested and fixed in 4% paraformaldehyde prior to scanning. Micro-CT scanning was conducted using a micro-CT system (PingSheng Medical Technology Co., Ltd., China). Reconstructed images of the cranial defect region were obtained in the coronal, sagittal, and transverse planes. A cylindrical region of interest (ROI) corresponding to the original defect area (5 mm in diameter) was selected for quantitative analysis. Bone regeneration within the defect was quantified using dedicated analysis software. Quantitative parameters, including bone volume (BV), bone volume fraction (BV/TV), trabecular number (Tb.N), trabecular thickness (Tb.Th), and trabecular separation (Tb.Sp), were calculated to assess new bone formation.

### Histological evaluation

At 4 and 8 weeks post-implantation, calvarial specimens were harvested for histological evaluation. The samples were fixed in 4% paraformaldehyde for 48 h and subsequently decalcified in 10% ethylenediaminetetraacetic acid (EDTA). After decalcification, specimens were dehydrated through graded ethanol, embedded in paraffin, and sectioned into 5 μm-thick serial sections for hematoxylin and eosin (H&E) and Masson’s trichrome staining. All stained sections were observed and imaged using an optical microscope (Nikon, Japan). To assess in vivo biosafety, major organs (heart, liver, spleen, lung, and kidney) were harvested at 8 weeks post-operation, fixed in paraformaldehyde, and subjected to H&E staining for histopathological evaluation.

### L929 biocompatibility evaluation

To evaluate the soft tissue wound healing performance of the hydrogels, L929 mouse fibroblasts (Sangon Biotech, Shanghai, China) were utilized in accordance with ISO 10,993 standards for initial biocompatibility screening. Cells were cultured in high-glucose DMEM supplemented with 10% fetal bovine serum (Gemini, USA) and 1% penicillin–streptomycin (Hyclone, USA) at 37 °C with 5% CO₂. Hydrogel-conditioned medium was prepared by incubating sterilized CH and rhBMP-2/CPC@CH hydrogels in complete culture medium. L929 cells were cultured in the conditioned medium, and cell viability was determined after 24 h using a Live/Dead cell staining kit (Yeasen Biotechnology, Shanghai, China). Cell proliferation was examined at 1 and 3 days using a CCK-8 assay (Yeasen Biotechnology, Shanghai, China).

### Antibacterial evaluation

The antibacterial activity of CH and rhBMP-2/CPC@CH hydrogels was investigated using Staphylococcus aureus (ATCC 29213) and Escherichia coli (ATCC 25922). Bacteria were cultured in Luria–Bertani (LB) medium at 37 °C to the logarithmic growth phase. Bacterial suspensions were incubated with different hydrogels, and optical density (OD₆₀₀) was measured at designated time points to generate growth curves. Bacterial viability on the hydrogel surfaces was evaluated by Live/Dead staining using a DMAO/PI bacterial staining kit (Beyotime Biotechnology, China). Bacteria recovered after hydrogel contact were serially diluted and plated on LB agar plates for colony-forming unit (CFU) counting after 24 h. Biofilm metabolic activity was further assessed using an MTT assay. All antibacterial experiments were performed under aseptic conditions.

### Statistical analysis

All quantitative data are presented as mean ± standard deviation. Statistical analysis was conducted using GraphPad Prism 10.0 software. Image quantification was performed using ImageJ software. Comparisons between multiple groups were analyzed using one-way or two-way analysis of variance (ANOVA) followed by Tukey’s post-hoc test for multiple comparisons, as appropriate. A value of *P* < 0.05was considered statistically significant.

## Results

### Characterization of rhBMP-2/CPC@CH hydrogels

**Fig. 1:**
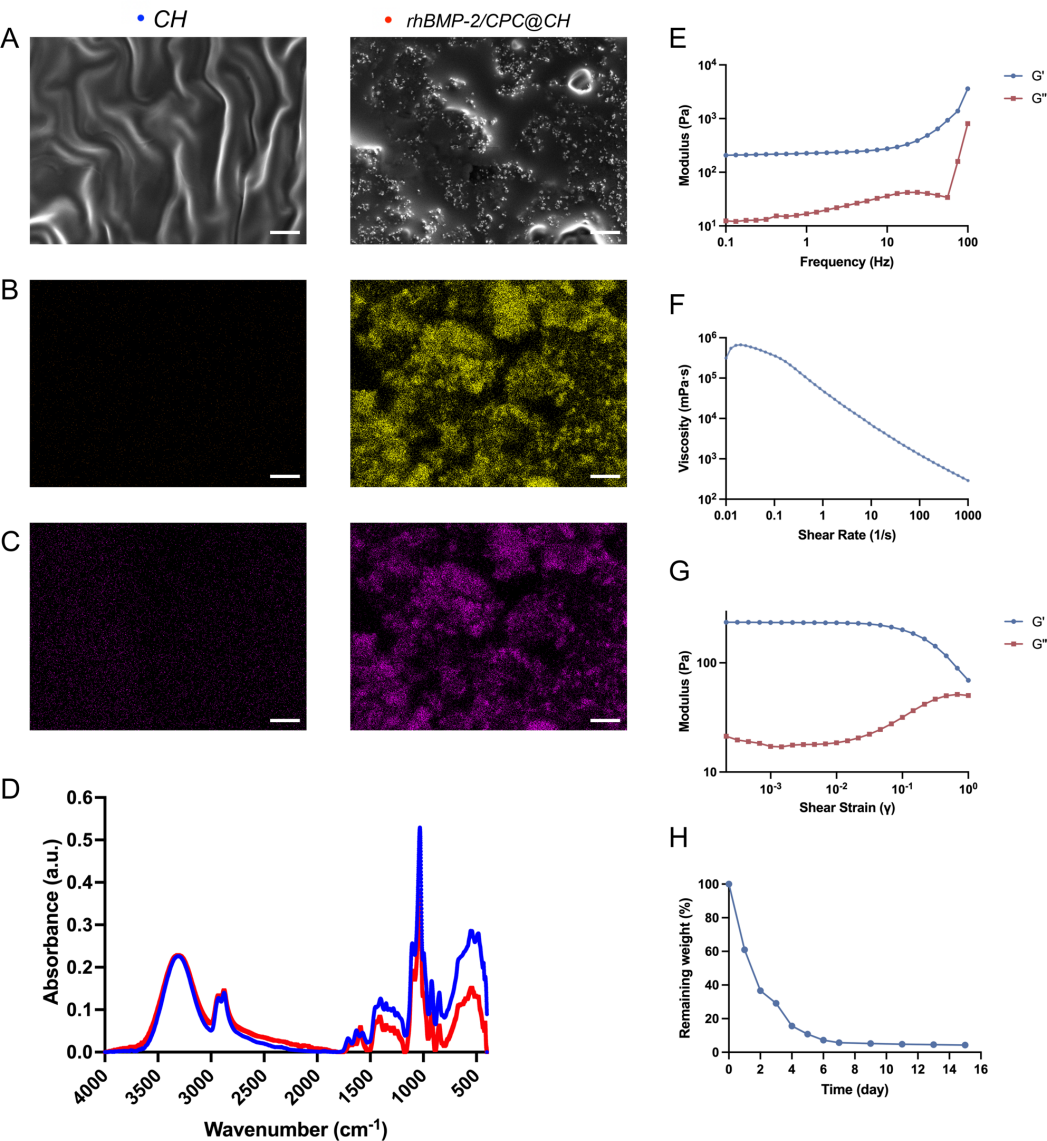
Characterization of CH and rhBMP-2/CPC@CH hydrogels.** A** SEM images of surface morphology (scale bar = 100 μm).** B**,** C** EDS mapping of (**B**) Calcium and (**C**) Phosphorus elements (scale bars = 100 μm).** D** FTIR spectra. (**E**) Frequency sweep results of rhBMP-2/CPC@CH hydrogel. **F** Shear rate-dependent viscosity curves of rhBMP-2/CPC@CH hydrogel. **G** Amplitude sweep results of rhBMP-2/CPC@CH hydrogel. **H** Enzymatic degradation profiles of rhBMP-2/CPC@CH hydrogel

SEM imaging revealed a smooth surface for the pure CH hydrogel. In contrast, the rhBMP-2/CPC@CH group showed a uniform distribution of CPC granules throughout the chitosan matrix, with no visible cracks or aggregation (Fig. 1A). EDS mapping confirmed the material composition, showing calcium (Ca) and phosphorus (P) signals concentrated at the granule sites (Fig. 1B, C). FTIR spectra presented characteristic peaks for both chitosan and CPC, verifying the successful synthesis of the composite (Fig. 1D). In rheological tests, the storage modulus (G′) consistently exceeded the loss modulus (G″), indicating a stable gel state (Fig. 1E). The hydrogel demonstrated shear-thinning behavior, with viscosity decreasing as the shear rate increased (Fig. 1F). Amplitude sweep analysis confirmed its self-healing capability, as the modulus rapidly recovered following high-strain structural disruption (Fig. 1G). The enzymatic degradation assay showed a rapid initial degradation phase followed by a transition to a stable state (Fig. 1H).

### In vitro biocompatibility and osteogenic differentiation

**Fig. 2:**
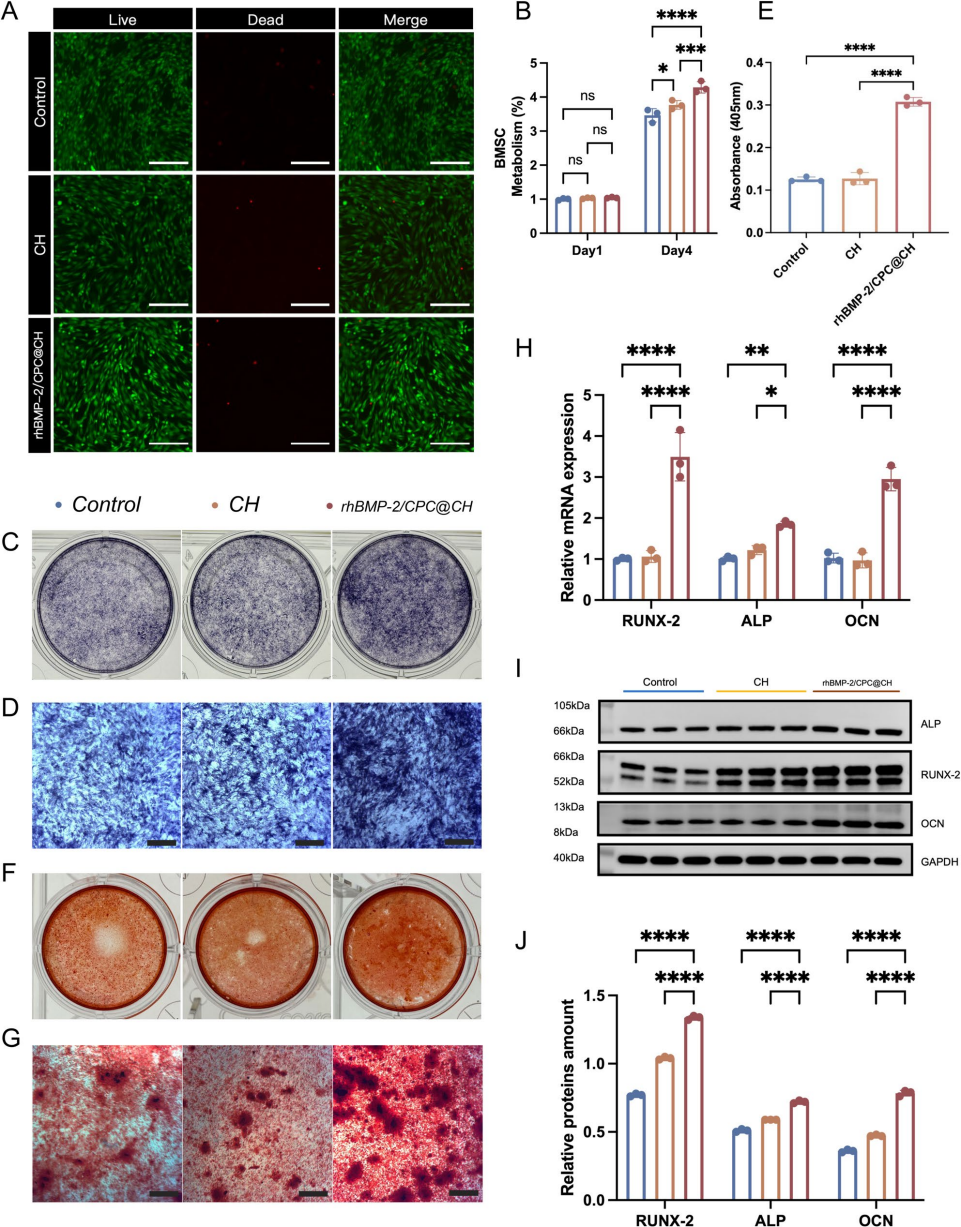
Cytocompatibility and osteogenic differentiation of BMSCs induced by hydrogels. **A** Representative Live/Dead staining images of BMSCs after 24 h of culture (scale bars = 200 μm). **B** BMSC proliferation evaluated by CCK-8 assay. **C**,** D** ALP staining and (**E**) quantitative ALP activity analysis after 14 days of induction (scale bars = 200 μm). **F**,** G** Alizarin Red S staining after 21 days (scale bars = 200 μm). **H** RT-qPCR analysis of *RUNX-2*, *ALP*, and *OCN* gene expression at day 10. **I** Western blot images and (**J**) corresponding quantitative analysis of RUNX-2, ALP, and OCN protein expression at day 14. **Data are presented as mean ± SD (*n* = 3); ns indicates no significant difference, *P < 0.05, **P < 0.01, ***P < 0.001, and ****P < 0.0001

Live/Dead staining at 24 h revealed robust BMSC viability across all groups with negligible cell death (Fig. 2A). Quantitatively, the CCK-8 assay indicated that the rhBMP-2/CPC@CH group significantly promoted cell proliferation compared to the Control and pure CH groups (Fig. 2B). In terms of osteogenic potential, the rhBMP-2/CPC@CH group exhibited the most intense ALP staining and significantly elevated ALP activity after 14 days of induction (Fig. 2C–E). This enhanced differentiation capacity was further confirmed by Alizarin Red S staining at day 21, which displayed a markedly higher density of large calcified nodules in the composite group (Fig. 2F, G). At the molecular level, RT-qPCR analysis at day 10 showed significant upregulation of osteogenic marker genes (*RUNX-2*, *ALP*, and *OCN*) in the rhBMP-2/CPC@CH group (Fig. 2H). Consistent with these transcriptional profiles, Western blot analysis at day 14 demonstrated significantly higher protein expression levels of RUNX-2, ALP, and OCN in the composite hydrogel group (Fig. 2I, J).

### In vivo bone regeneration and systemic safety evaluation

**Fig. 3:**
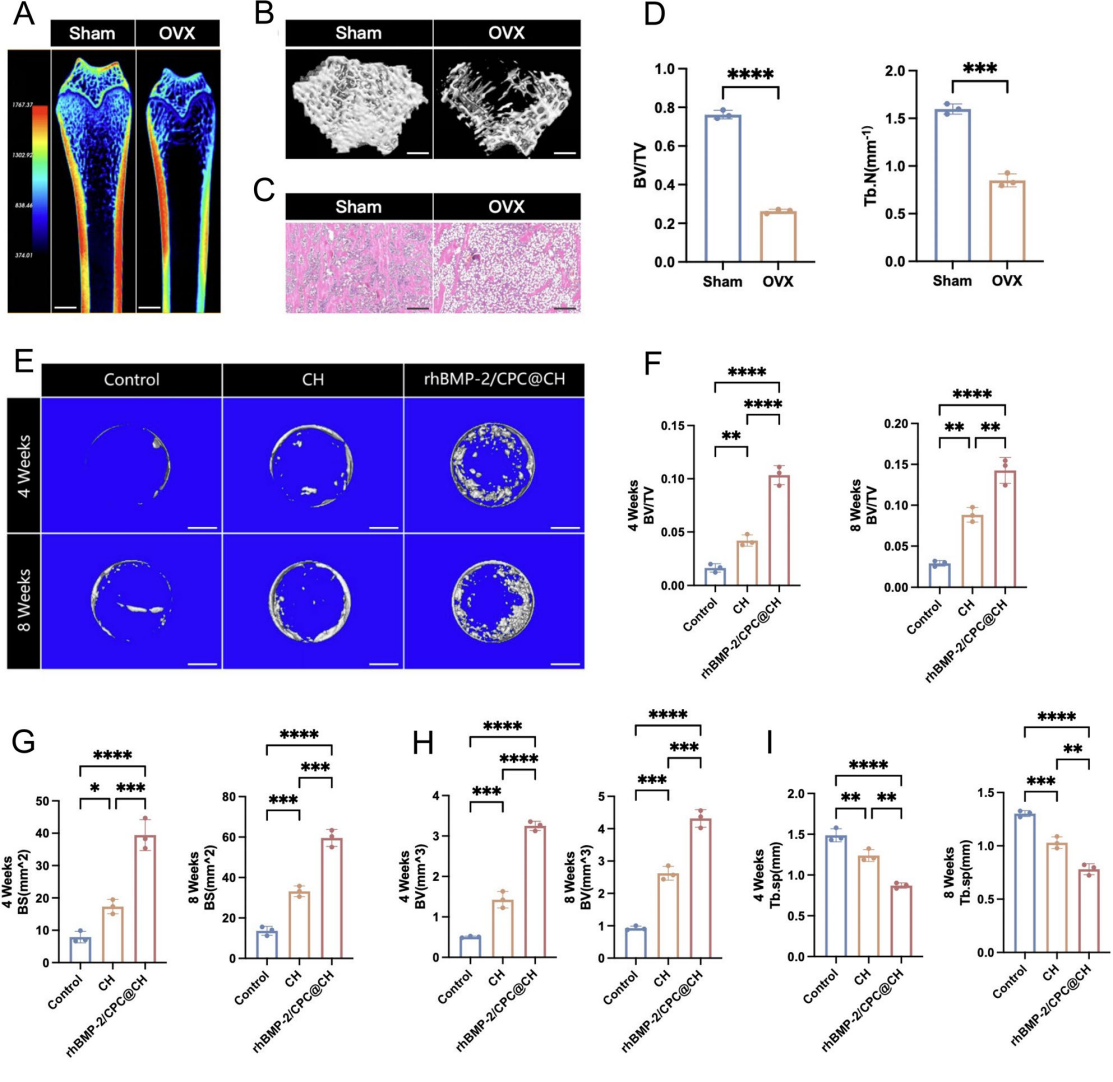
Validation of the OVX model and radiological assessment of cranial bone repair. **A–D** Validation of the osteoporotic model: (**A**) 3D micro-CT femur reconstructions in the coronal plane (scale bar = 2 mm), **B** 3D micro-CT femur reconstructions in the transverse plane (scale bar = 2 mm), **C** H&E staining of the femur (scale bar = 200 μm), and (**D**) quantitative analysis of femur BV/TV and Tb.N. **E** Representative 3D micro-CT reconstructions of cranial defects at 4 and 8 weeks (scale bar = 5 mm). **F–I** Quantitative analysis of regenerated bone parameters: (**F**) BV/TV, (**G**) bone surface (BS), (**H**) bone volume (BV), and (**I**) trabecular separation (Tb.Sp). **Data are presented as mean ± SD (*n* = 3); *P < 0.05, **P < 0.01, ***P < 0.001, and ****P < 0.0001

Six weeks post-ovariectomy, micro-CT reconstructions of the femur revealed a significant reduction in bone mineral density and a fragmented trabecular network in the OVX group (Fig. 3A, B). Quantitative analysis confirmed substantial decreases in BV/TV and Tb.N compared to the Sham group (Fig. 3D). H&E staining further exhibited disordered bone trabeculae and extensive adipocyte infiltration within the marrow cavity (Fig. 3C), validating the successful establishment of the osteoporotic model. Following cranial defect surgery, reconstructed 3D images showed minimal bone formation in the control group, whereas the rhBMP-2/CPC@CH group displayed prominent new bone deposition at the defect margins at 4 weeks, and the defects were nearly closed by 8 weeks (Fig. 3E). Quantitative parameters corroborated these observations, as the rhBMP-2/CPC@CH group exhibited significantly higher BV/TV, BS, and BV, along with a marked reduction in Tb.Sp, compared to both the Control and CH groups at all time points (Fig. 3F-I).

**Fig. 4:**
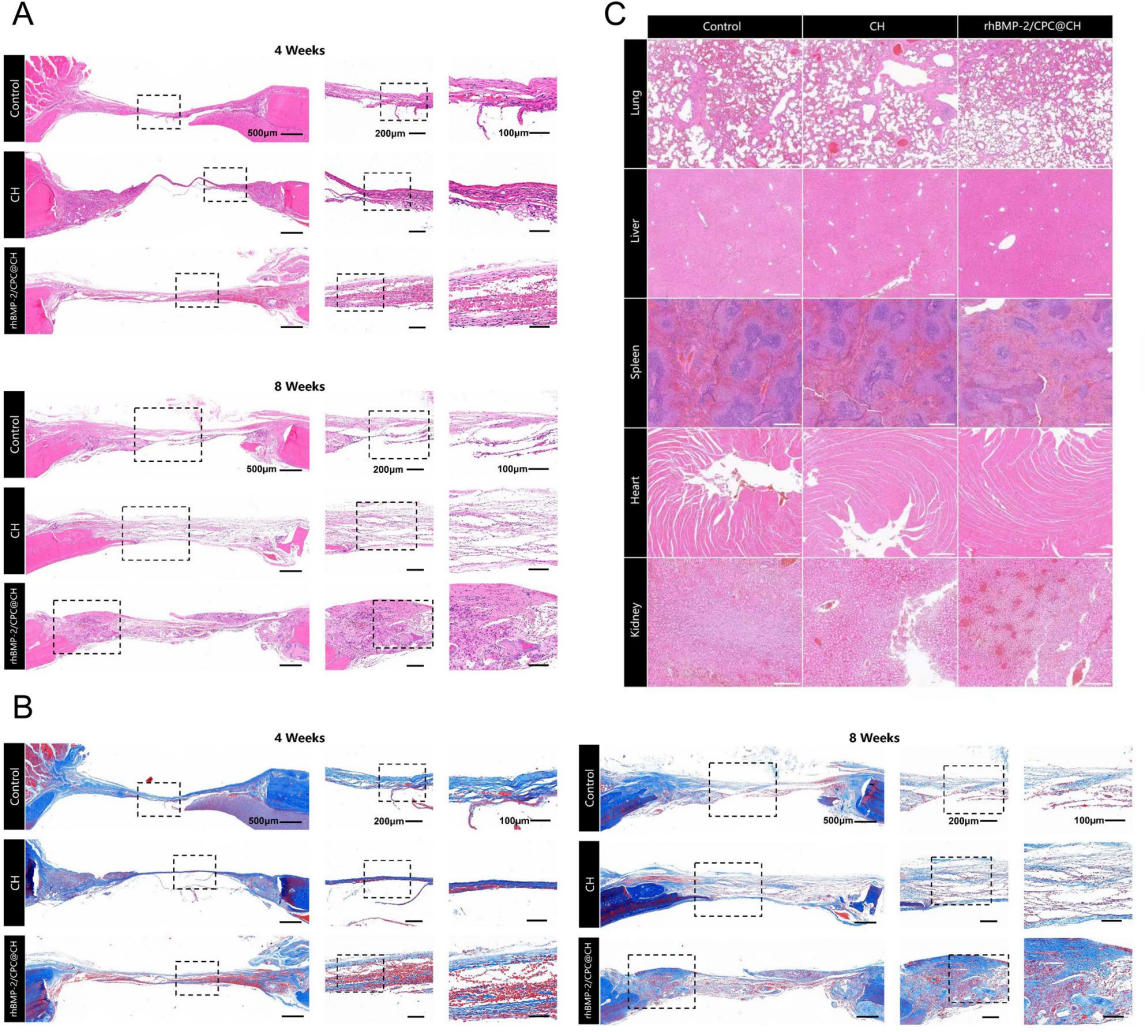
Histological evaluation of bone regeneration and systemic safety.** A** H&E staining and (**B**) Masson’s trichrome staining of the cranial defect sites at 4 and 8 weeks post-implantation (scale bars as indicated in the figures). **C** H&E-stained sections of the heart, liver, spleen, lung, and kidney at 8 weeks post-operation showing no systemic toxicity (scale bar = 500 μm). Data are presented as mean ± SD (*n* = 3)

H&E staining at 4 and 8 weeks revealed that the rhBMP-2/CPC@CH group was occupied by organized trabecular bone and dense extracellular matrix, while the control group remained primarily filled with fibrous tissue (Fig. 4A). Masson’s trichrome staining showed more extensive blue-stained collagen and red-stained mature bone in the rhBMP-2/CPC@CH group, indicating enhanced mineralization (Fig. 4B). Additionally, H&E sections of major organs showed no pathological alterations or inflammatory infiltration across all groups at 8 weeks (Fig. 4C), confirming the systemic biosafety of the hydrogels.

### L929 biocompatibility and antibacterial activity

**Fig. 5:**
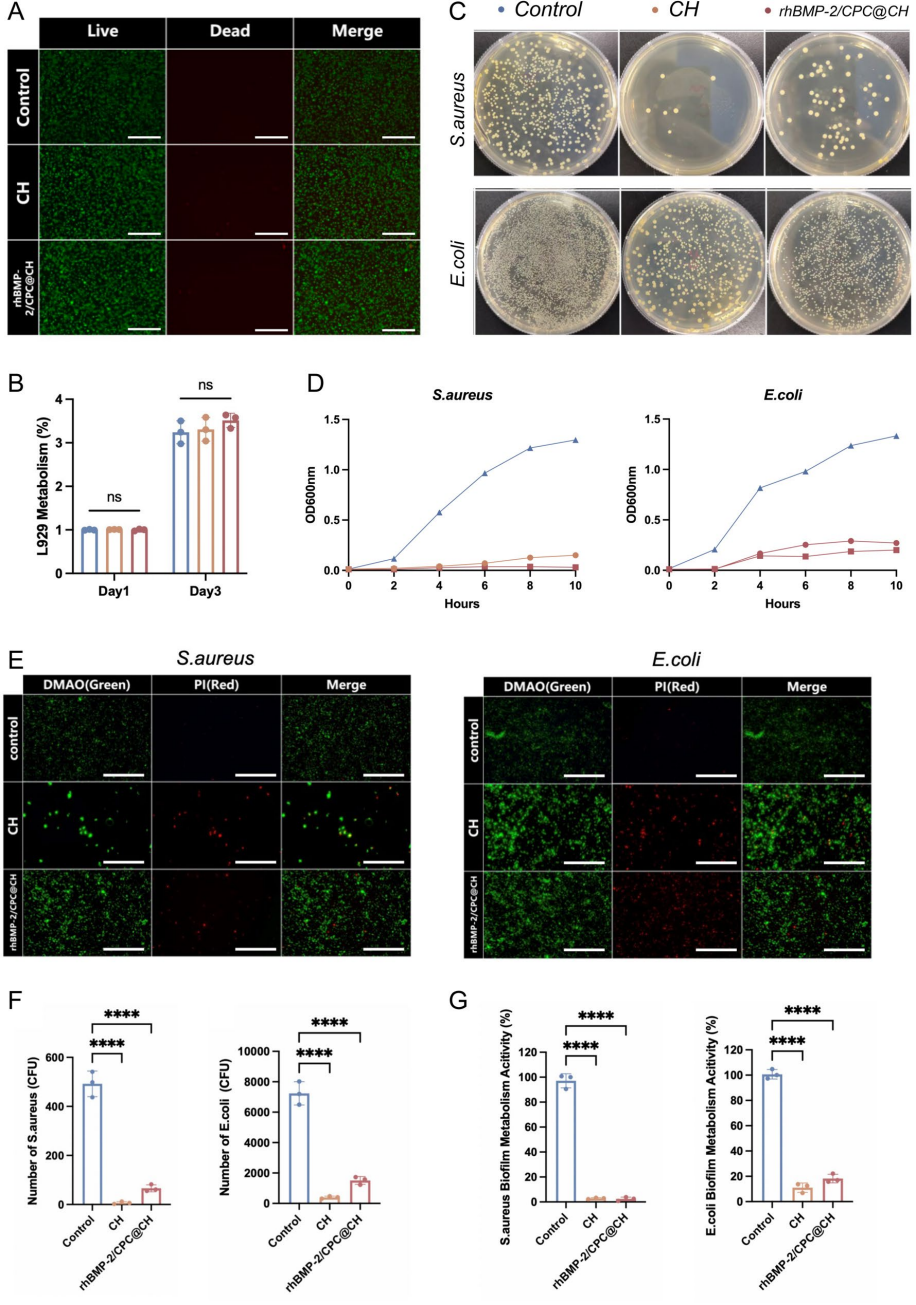
Cytotoxicity, proliferation of L929 cells and antibacterial performance of the hydrogels. **A** Live/dead staining of L929 cells after co-culture with Control, CH, and rhBMP-2/CPC@CH hydrogels (green: live cells; red: dead cells; scale bar = 200 μm). **B** Metabolic activity of L929 cells at 1 and 3 days evaluated by CCK-8 assay (*ns*, *P* > 0.05). **C** Representative images of bacterial colonies on LB agar plates after co-culture with different hydrogels. **D** Bacterial growth curves of *S. aureus* and *E. coli* over 10 h. **E** Live/dead staining of bacteria on the surface of hydrogels (green: live bacteria; red: dead bacteria; scale bar = 50 μm). **F** Quantitative analysis of colony-forming units (CFUs) for *S. aureus* and *E. coli*. **G** Metabolic activity of bacterial biofilms on the hydrogel surfaces evaluated by MTT assay. Data are presented as mean ± SD (*n* = 3), ****P < 0.0001 compared with the control group

Fluorescence imaging demonstrated that L929 cells maintained a high survival rate, characterized by extensive green staining and a scarcity of red signals across all groups (Fig. 5A). Cell morphology remained consistent across the Control, CH, and rhBMP-2/CPC@CH groups. Quantitative metabolic activity analysis revealed no significant differences between the hydrogel groups and the control at 1 and 3 days (Fig. 5B), indicating that the materials possess excellent cytocompatibility and do not inhibit cell proliferation. In addition to biocompatibility, the hydrogels demonstrated significant antibacterial properties. Bacterial growth curves (Fig. 5D) demonstrated that OD600 values for the CH and rhBMP-2/CPC@CH groups remained at a sustained low level over 10 h, in contrast to the rapid proliferation observed in the control group. These findings were corroborated by spread plate images (Fig. 5C) and corresponding colony counts (Fig. 5F), where both hydrogel groups exhibited a significant reduction in CFUs. Live/dead bacterial staining (Fig. 5E) further revealed a substantial increase in red fluorescence on the hydrogel surfaces, and the metabolic activity of bacterial biofilms was significantly lower in the hydrogel groups than in the control (Fig. 5G).

## Discussion

The restoration of osteoporotic bone defects is a formidable challenge due to the systemic disruption of bone metabolism and the compromised regenerative microenvironment [16, 17]. Traditional grafting materials often lack sufficient intrinsic osteoinductivity to initiate effective regeneration under these conditions [18]. This study developed an injectable rhBMP-2/CPC@CH composite hydrogel to address these issues. The system functions as a shape-adaptive filler and a bioactive modulator, utilizing a thermosensitive chitosan (CH) matrix for in situ gelation and calcium phosphate cement (CPC) microspheres for osteoconductive support. By incorporating rhBMP-2, the hydrogel overcomes the functional inertia of osteoporotic BMSCs, establishing a microenvironment conducive to regeneration.

Injectable scaffolds require a balance between fluidity during administration and structural stability post-implantation. SEM and EDS mapping confirmed that CPC granules were uniformly sequestered within the chitosan matrix without aggregation. This structural homogeneity underpins the material’s stable gel state under low strain, essential for maintaining the spatial distribution of bioactive payloads. The observed shear-thinning and self-healing behaviors facilitate clinical translation, allowing the material to flow through narrow needles and regain mechanical integrity to prevent the wash-out of active components [19]. Beyond the ease of delivery, the capacity for in situ adaptation to complex defect geometries provides a distinct advantage over rigid, pre-fabricated scaffolds. By conforming precisely to irregular voids, the hydrogel establishes seamless interface contact with the host bone while significantly minimizing surgical trauma, which is particularly beneficial for elderly osteoporotic patients.

FTIR analysis confirmed the formation of intermolecular hydrogen bonds between the matrix components, indicated by shifts in the hydroxyl, amino, and amide I regions. This chemical entrapment transforms the hydrogel into a sustained-release reservoir, protecting the bioactivity of rhBMP-2 and avoiding the adverse effects of burst release [20, 21]. The release profile is synchronized with the biphasic enzymatic degradation observed in this study. As the chitosan matrix is gradually replaced by host tissue through crawling substitution, the sequestered CPC remains to provide a stable template for mineralized bone ingrowth [22, 23].

BMSCs function as the primary seed cells for bone regeneration. CCK-8 and Live/Dead staining confirmed that BMSCs maintained high viability and metabolic activity on the rhBMP-2/CPC@CH hydrogel. The biomimetic, interconnected architecture facilitates cell adhesion, migration, and nutrient transport [24]. Supported by this microenvironment, the hydrogel demonstrated a potent capacity to drive osteogenic differentiation. This was validated by the upregulation of ALP activity and the extensive deposition of calcium nodules. The scaffold significantly enhanced the expression of RUNX-2, ALP, and OCN, indicating the successful initiation of the osteogenic transcription program and osteoblast maturation.

The BMP-2/Smad signaling pathway drives this osteogenic enhancement. rhBMP-2 binds to transmembrane receptors and triggers the phosphorylation of Smad1/5/8, with ALP acting as a direct downstream target [25]. The efficacy of this signaling is preserved by the material’s dual-retention capability. Electrostatic interactions between positively charged chitosan and weakly negatively charged rhBMP-2 create an affinity-based reservoir [26]. Additionally, the physical steric hindrance of the network prolongs the protein diffusion path [27]. This mechanism mitigates burst release, ensuring the sustained biological stimulus necessary for osteogenic efficiency [28].

The OVX model was employed to replicate the systemic challenges and impaired healing typical of postmenopausal osteoporosis, providing a rigorous test for the hydrogel’s efficacy. In the current study, the rhBMP-2/CPC@CH hydrogel yielded superior bone volume fraction and trabecular thickness in the OVX rat calvarial defect model. Histological assessment revealed new bone bridging and organized collagen deposition, whereas the pure CH group displayed limited formation. Zhao et al. demonstrated that amino and hydroxyl groups in chitosan facilitate initial mineral interactions, explaining the moderate benefit in the pure CH group [29]. Previous studies indicated that chitosan degradation products modulate the local microenvironment to support early healing [30–32]. The robust regeneration in the composite group implies a synergistic mechanism: the CPC scaffold provides a template for host cell recruitment and crawling substitution [33], while the chitosan network prevents growth factor washout [19]. This counteracts the functional inertia of osteoporotic BMSCs, as prolonged rhBMP-2 exposure overrides the estrogen deficiency-induced signaling deficit [34]. This synergistic regeneration involves CPC granules acting as mineral templates, the chitosan matrix providing antibacterial defense, and rhBMP-2 delivering osteoinductive stimuli.

Infection-induced chronic inflammation accelerates bone loss in osteoporotic patients [35]. Bacterial biofilms enhance drug resistance and lead to recalcitrant infections [36–38]. Since surgical wounds often serve as the primary entry route for pathogens, preserving the soft tissue barrier is critical for preventing invasion. In this study, L929 fibroblasts confirmed the soft tissue safety of the hydrogel, showing high cell viability and indicating no negative effect on wound healing. Regarding infection control, the hydrogel demonstrated comprehensive activity against *S. aureus* and *E. coli*. Bacterial growth curves, plate counts, DMAO/PI staining, and MTT assays verified the inhibition of planktonic growth, adhesion, and biofilm maturation. This efficacy is likely attributed to the intrinsic polycationic nature of chitosan, where amino groups disrupt bacterial membranes via electrostatic interactions [39]. Although the antibacterial performance in the composite group was slightly reduced compared to pure chitosan due to potential charge neutralization, it retains sufficient potency to provide a dual-protective microenvironment.

## Conclusion

This study developed an injectable rhBMP-2/CPC@CH hydrogel to address the challenge of osteoporotic bone defects by leveraging the synergistic advantages of rhBMP-2, CPC, and chitosan. The outcomes reveal that this hydrogel, characterized by facile preparation and superior injectability, integrates excellent biocompatibility, potent osteogenic activity, and intrinsic antibacterial performance to facilitate robust bone regeneration within a protective microenvironment. These results underscore the potential clinical utility of the rhBMP-2/CPC@CH hydrogel as a promising minimally invasive therapeutic strategy for the clinical repair of osteoporotic bone defects, providing a foundational basis for future clinical trials and applications.

## Supplementary Information

Supplementary Material 1.

## Data Availability

The datasets generated and/or analyzed during the current study are available from the corresponding author on reasonable request.

## Abbreviations

OP: Osteoporosis
CPC: Calcium phosphate cement
CH: Chitosan
rhBMP-2: Recombinant human bone morphogenetic protein-2
BMSCs: Bone marrow mesenchymal stem cells
OVX: Ovariectomized
SEM: Scanning electron microscopy
EDS: Energy-dispersive spectroscopy
FTIR: Fourier transform infrared spectroscopy
