# Supplementary material for: Injectable rhBMP-2-loaded calcium phosphate cement and chitosan composite hydrogel for the repair of osteoporotic bone defects
Source: BMC Musculoskelet Disord. 2026 Mar 9;27:320. doi: 10.1186/s12891-026-09717-w (PMC13085745; doi:10.1186/s12891-026-09717-w)
Supplement: Supplementary file 1 — Supplementary Material 1. [file 12891_2026_9717_MOESM1_ESM.pdf]

Supplementary Materials

Title: Injectable rhBMP-2-loaded Calcium Phosphate Cement and Chitosan Composite Hydrogel for the Repair of Osteoporotic Bone Defects

Authors: Minglei Cai [1*], Jian Huang [2*], Fulong Zhong [3*], Bowen Lai [2], Jing Wang [1#], Tielong Liu [1#]

Figure 2I **Original**

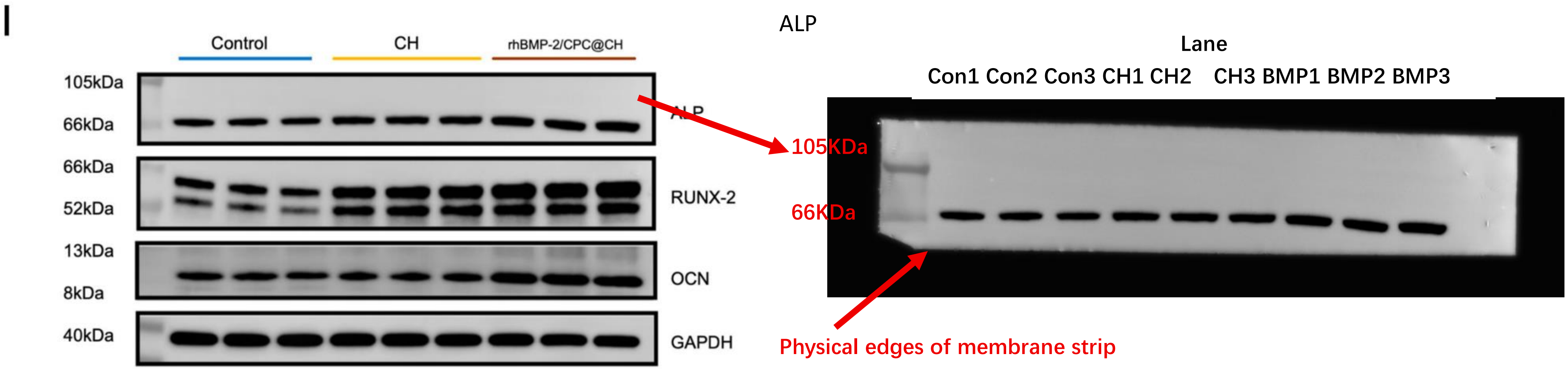

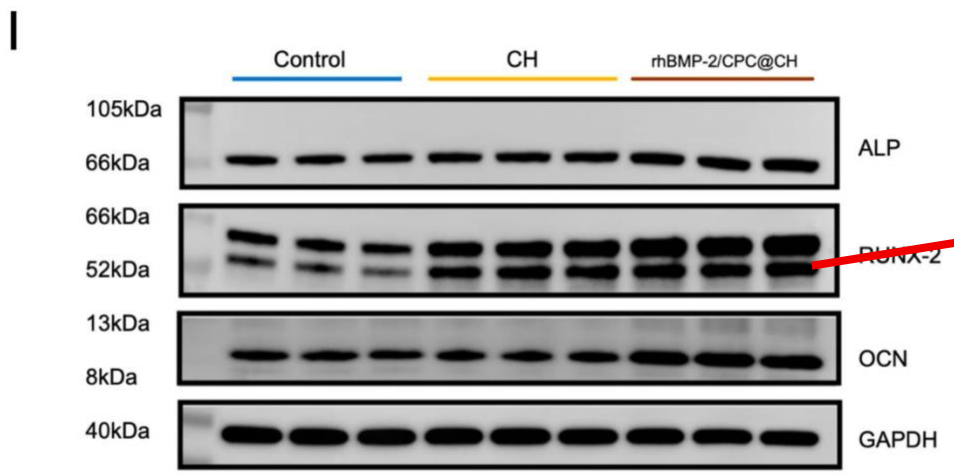

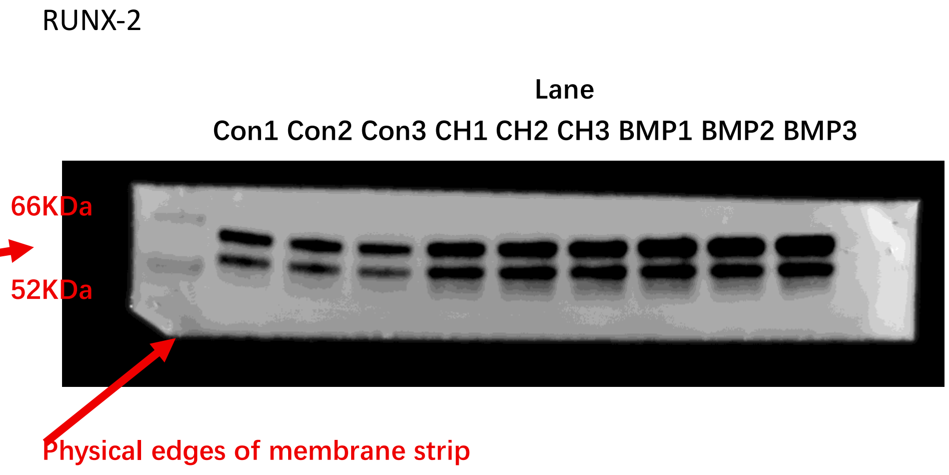

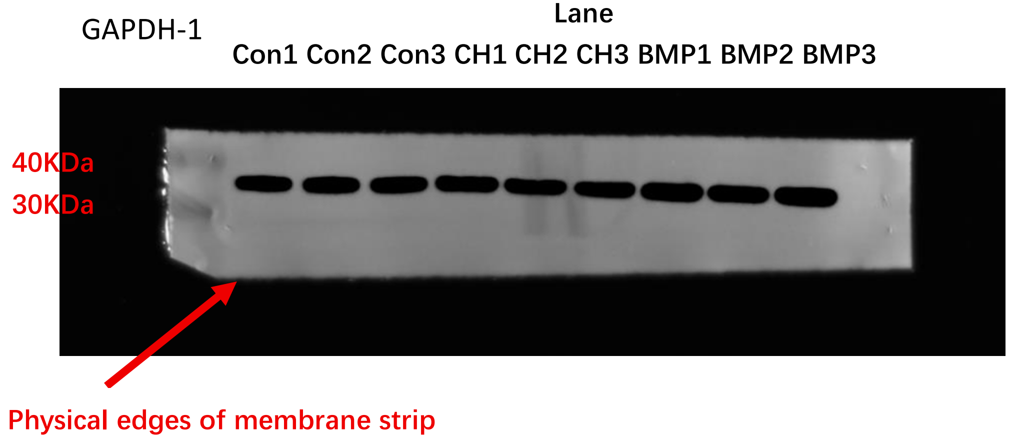

Group 1: RUNX2 and ALP were analyzed on one set of membranes with GAPDH-1 as the internal control.

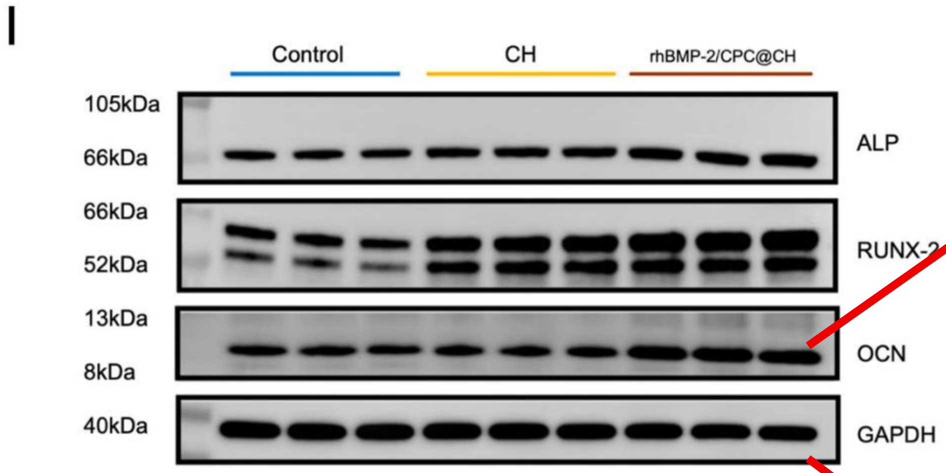

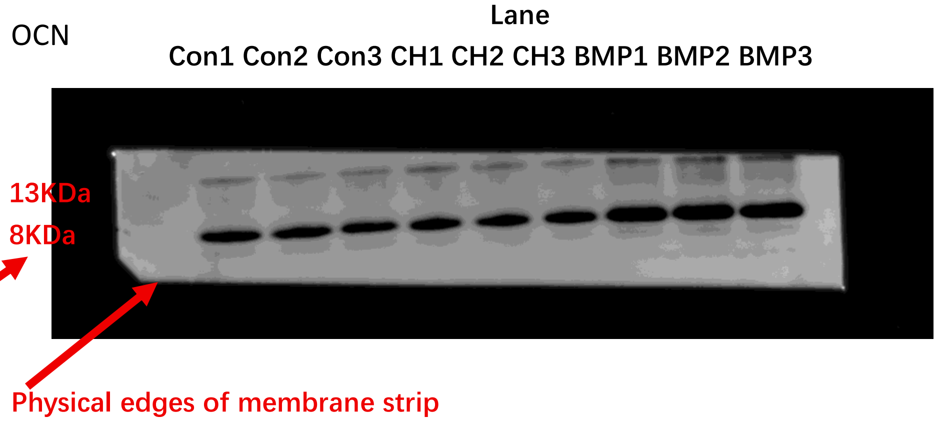

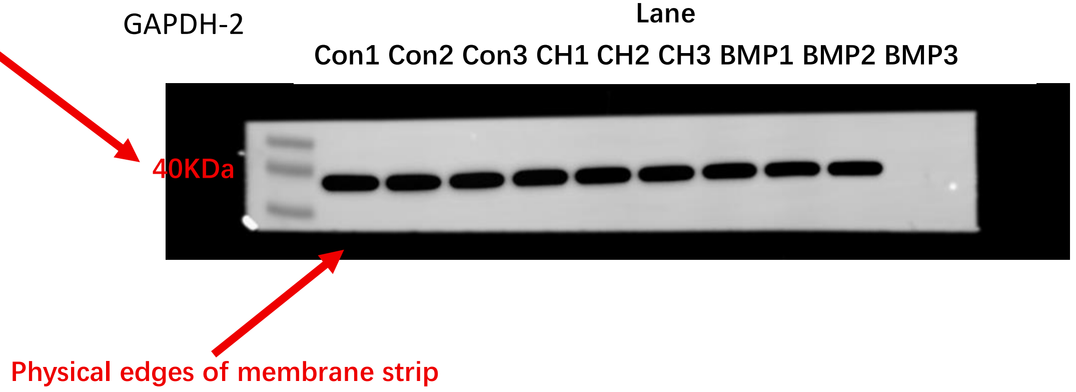

Group 2: OCN were analyzed on one set of membranes with GAPDH-2 as the internal control.
